# Fatal outcome after insufficient spine fixation for pyogenic thoracic spondylodiscitis: an imperative for 360° fusion of the infected spine

**DOI:** 10.1186/1754-9493-3-4

**Published:** 2009-02-25

**Authors:** Michael A Flierl, Kathryn M Beauchamp, Gene E Bolles, Ernest E Moore, Philip F Stahel

**Affiliations:** 1 Department of Orthopaedic Surgery, Denver Health Medical Center, University of Colorado School of Medicine, 777 Bannock Street, Denver, CO 80204, USA; 2 Division of Neurosurgery, Denver Health Medical Center, University of Colorado School of Medicine, 777 Bannock Street, Denver, CO 80204, USA; 3 Department of Surgery, Denver Health Medical Center, University of Colorado School of Medicine, 777 Bannock Street, Denver, CO 80204, USA

## Abstract

**Background:**

Pyogenic spondylodiscitis represents a potentially life-threatening condition. Due to the low incidence, evidence-based surgical recommendations in the literature are equivocal, and the treatment modalities remain controversial.

**Case presentation:**

A 59 year-old patient presented with a history of thoracic spondylodiscitis resistant to antibiotic treatment for 6 weeks, progressive severe back pain, and a new onset of bilateral lower extremity weakness. Clinically, the patient showed a deteriorating spastic paraparesis of her lower extremities. An emergent MRI revealed a kyphotic wedge compression fracture at T7/T8 with significant spinal cord compression, paravertebral and epidural abscess, and signs of myelopathy. The patient underwent surgical debridement with stabilization of the anterior column from T6–T9 using an expandable titanium cage, autologous bone graft, and an anterolateral locking plate. The patient recovered well under adjunctive antibiotic treatment. She presented again to the emergency department 6 months later, secondary to a repeat fall, with acute paraplegia of the lower extremities and radiographic evidence of failure of fixation of the anterior T-spine. She underwent antero-posterior revision fixation with hardware removal, correction of kyphotic malunion, evacuation of a recurrent epidural abscess, decompression of the spinal canal, and 360° fusion from T2–T11. Despite the successful salvage procedure, the patient deteriorated in the postoperative phase, when she developed multiple complications including pneumonia, acute respiratory distress syndrome, bacterial meningitis, abdominal compartment syndrome, followed by septic shock with multiple organ failure and a lethal outcome within two weeks after revision surgery.

**Conclusion:**

This catastrophic example of a lethal outcome secondary to failure of anterior column fixation for pyogenic thoracic spondylodiscitis underlines the notion that surgical strategies for the infected spine must be aimed at achieving absolute stability by a 360° fusion. This aggressive – albeit controversial – concept allows for an adequate infection control by adjunctive antibiotics and reduces the imminent risk of a secondary loss of fixation due to compromises in initial fixation techniques.

## Introduction

Spinal infections are rare and the annual incidence of spondylodiscitis is estimated to be about 2.4/100,000 person-years [1]. However, spondylodiscitis is associated with high morbidity and mortality rates [2]. While only 0.2–4% of all spinal procedures result in postoperative discitis [3], spinal infections after surgery can become a life-threatening event to the patient [4]. In its early stages, spondylodiscitis usually responds favorably to antibiotic therapy [5]. If non-surgical treatment for more than 6 weeks fails, or if patients deteriorate secondary to paraspinal abscesses, sepsis, spinal deformities due to destruction of the endplates or progressive neurological impairment, operative treatment must be considered [6]. Most recommendations for surgical treatment of spinal infections are largely based on retrospective case series or case reports and remain inconsistent [7,8]. In particular, the use of metal implants at a septic focus has traditionally been rigorously avoided since pathogen adhesion to the implants present a matter of concern [9]. More recently, this notion has been challenged by reports in the literature which presented favorable outcomes using titanium cages in the surgical management of spondylodiscitis [10-12]. Whether this paradox of a distinct management strategy for osteomyelitis in the spine as opposed to infected peripheral long bones, e.g. the tibia, is related to the solid immunological properties of the highly vascularized spine and adequate soft tissue coverage, remains a topic of speculation [10]. Despite these recent promising reports on the successful aggressive debridement and spinal stabilization procedures [10-14], the adequate surgical treatment modalities for the infected spine has not been unequivocally defined until present.

In the present paper, we present the catastrophic example of a 59-year old patient with inadequate initial fixation for thoracic spondylodiscitis, with a fatal outcome two weeks after revision surgery with 360° thoracic spine fusion.

## Case report

**Figure 1:**
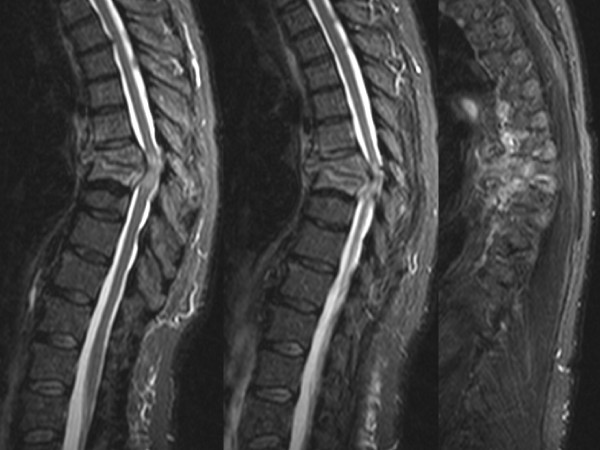
**Initial MRI obtained on the first day in the emergency department**. STIR sagittal views show the extent of kyphosis at T7/T8 with vertebral body destruction due to pyogenic thoracic spondylodiscitis, spinal canal compression, anterior paravertebral and epidural abscess, and evidence of myelopathy at the T7/T8 level.

**Figure 2:**
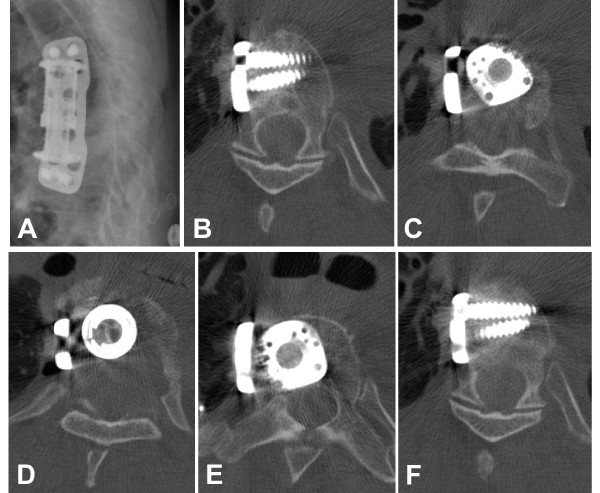
**Status post anterior debridement through a right-side anterolateral thoracotomy, T7 and T8 corpectomy, anterior spinal canal decompression, and anterior fusion T6 through T9 by expandable cage, autologous bone graft, and anterolateral locking plate**. Panel A shows the adequate restoration of the sagittal profile of the thoracic spine, on plain X-rays. The axial CT views demonstrate the adequate locking head screw placement of the anterolateral plate (panels B, F) and adequate expandable cage placement with spinal canal clearance (panels C, D, E).

A 59-year-old lady with a known history of intravenous heroin abuse, arterial hypertension, and chronic hepatitis C, presented to our emergency department with severe thoracic back pain and progressive spastic paraparesis of her lower extremities. The patient had a past history of aT7/T8 spondylodiscitis with epidural abscess, and failed conservative treatment by intravenous antibiotics at an outside institution, where the patient had been initially treated with nafcillin 10 g i.v. for 6 weeks. Due to unsuccessful eradication, the antibiotic regimen was then adapted changed to cefazolin 1 g i.v. twice daily for 6 weeks. An emergent MRI on the day of admission to our emergency departments showed a kyphotic wedge compression fracture at T7/T8 with significant spinal cord compression and myelopathy (Figure 1). She was taken to surgery the same day for a right-side anterolateral thoracotomy, radical surgical debridement, anterior corpectomy T7 and T8, discectomy T6/T7, T7/T8 and T8/T9 and anterior spinal canal decompression, prevertebral and epidural abscess evacuation. Spinal stabilization from T6 through T9 (Figure 2) was performed by vertebral body replacement using an expandable titanium cage (Synthes Synex^® ^cage), autologous bone graft, and an anterolateral locking plate system (Synthes). There were no intra-/perioperative complications and the patient tolerated the surgical procedure well. Her postoperative course was uneventful, and the neurological impairment recovered within two weeks. She was fitted in an adjunctive TLSO brace and discharged on day 12 after clearance by physical and occupational therapy. Intravenous antibiotics were adjusted according to the intraoperative culture results and continued through a Hohn catheter on an outpatient basis with vancomycin 1 g i.v. per day for 6 weeks The patient followed up in clinic at regular intervals, showing an uneventful recovery with progressive ambulation, decreased back pain, and well-healed surgical wounds without any signs of a residual infection.

**Figure 3:**
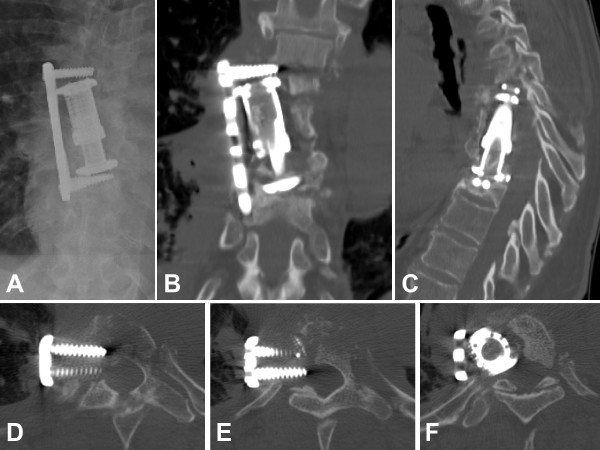
**Failure of the initial anterior fixation at 6 months after surgery, secondary to repeat falls**. Conventional anteroposterior X-ray (panel A) and coronal CT reconstruction (panel B) demonstrate the loss of fixation of the anterolateral locking plate and of the expandable cage, which are pulled out at the upper segment in T6. The sagittal CT reconstruction (panel C) demonstrates the kyphotic malunion cephalad to the expandabale cage. The axial CT views (panels D-F) shows the laterally pulled out locking head screws (D, E) and cage (F) at T6.

Six months after the procedure, she presented again to the emergency department, secondary to a repeat fall, with clinical signs of acute paraplegia of the lower extremities. Emergent radiographic evaluation by conventional films and CT scan revealed a failure of fixation of the anterior thoracic spine, with a cranial pull-out of the cage and locking plate in the coronal plane, and kyphotic malunion in the sagittal plane (Figure 3). The patient was taken back to the OR for revision surgery the next day.

**Figure 4:**
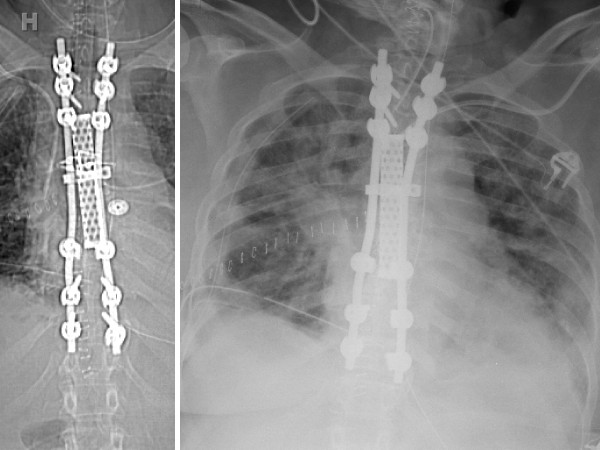
**Postoperative CT scout (A) and chest X-ray (B) after 360° revision fixation by posterior instrumentation T2–T11 and anterior PMMA/Tobramycin mesh cage placement**.

A posterior instrumentation was performed from T2 through T11 (Stryker Xia^® ^polyaxial internal fixator system) with correction of the kyphotic malunion and posterolateral bone grafting. The failed anterior fixation was revised through the previous right-side anterolateral thoracotomy, by removal of the failed expandable cage and anterolateral locking plate, revision debridement of a recurrent epidural abscess, and revision fixation from T4 through T9 using a titanium mesh cage (Stryker, V-Boss^® ^cage) filled with PMMA/Tobramycin cement (Figure 4). Despite the successful salvage procedure, the patient deteriorated in the postoperative phase in the surgical intensive care unit (SICU). She developed bacteremia, meningitis, sepsis and eventually a septic shock. Blood cultures were positive for Methicillin-sensitive *S. aureus*, and the patient developed a *P. aeruginosa *pneumonia, leading to acute respiratory distress syndrome (ARDS). A spinal tap further revealed positive cerebrospinal fluid (CSF) cultures for *E. coli*, implying a gram-negative bacterial meningitis. Intravenous antibiotic therapy was continued and modified according to the culture sensitivity testing with vancomycin 1 g i.v. per day Standard supportive SICU care was continued for management of ARDS and septic complications. Ultimatively, the patient developed a secondary abdominal compartment syndrome which led to impaired ventilatory capacity and the requirement for an emergent decompressive laparotomy. Within two weeks of spinal revision surgery, the patient succumbed to these postoperative complications, as a consequence of refractory septic shock with multiple organ failure.

## Discussion

The primary goal of surgical treatment of spondylodiscitis is the radical debridement of any infectious and necrotic tissues [13]. However, extensive debridement of the anterior column of the spine, which is known to be predominantly involved in vertebral osteomyelitis [15], often creates large defects that adversely affect spinal stability. While structural bone autografts represent the "gold standard" for reconstruction of these anterior column defects [7], harvest of large autografts is limited and is associated with considerable donor site morbidity [16]. Titanium mesh cages have been demonstrated to be a viable alternative for bridging large anterior defects [10-12], despite the traditional concept that metal implants are not to be implanted in sites of active infections.

Another "key" component of successful surgical treatment of spondylodiscitis is restoration of spinal anatomy and stability. Yet, there is currently a lack of consensus in the pertinent literature regarding the appropriate fixation modalities. The question of whether isolated anterior, isolated posterior, or combined antero-posterior fixation strategies represent the adequate therapy of choice remain a topic of debate. While some authors consider isolated anterior stabilization for single-level or larger defects an adequate treatment strategy [17], other groups advocate the exclusive posterior fixation with percutaneous drainage of anterior abscesses [18]. A recent retrospective study analyzed the outcome in 24 consecutive patients treated by spinal fusion for pyogenic spondylodiscitis, either by "stand-alone" anterior strut grafting, or by combined anterior interbody fusion with posterior instrumented fixation [7]. No recurrences of infection was described in either group, However, the authors found an increased incidence of complications related to the exclusive anterior bone graft fixation, compared to the antero-posterior, 360° fixation group [7]. In a similar study published in the German literature [19], the authors advocated a single-stage procedure of debridement and instrumented fusion for pyogenic spondylodiscitis, either by exclusive anterior or combined antero-posterior 360° fusion. No recurrence of infection was noted in either group during a follow-up period of 18 months [19]. The group of Christoph Heyde from Berlin has recently provided convincing evidence on safety and efficacy of the "radical" concept of surgical debridement with antero-posterior 360° fusion for pyogenic spondylodiscitis, based on multiple publications in the peer-reviewed literature [10,11,13]. The question of whether such severely ill patients are best treated by an aggressive "all-in-one" 360° procedure, or whether it is safer to postpone the individual procedures in terms of a staged concept of posterior and anterior spinal fusion, remains controversial [8,10-12,14].

Patients with spondylodiscitis frequently present with multiple co-morbidities [20,21] In the present case, our 59-year old patient had a long-standing history of hepatitis C, arterial hypertension, and intravenous drug abuse. The latter has been previously identified as an independent risk factor for spinal infection [20]. Given the significant co-morbidities, it remains a matter of speculation whether the patient presented in this case report may have survived the severe spine infection (Figure 1) by an initial, aggressive all-in-one 360° fusion, would have likely decreased the risk of a secondary loss of fixation (Figure 3) attributed to a reduced spinal stability by exclusive fixation of the anterior column (Figure 2). Due to the patient's i.v. drug addiction, repeated falls may have been anticipated, and the revision surgery secondary to loss of fixation, which initiated the cascade of subsequent postoperative complications, leading to the ultimate lethal outcome, may have been avoided. Collectively, the root causes for failed index surgery in this case are likely multiple. Persistent chronic systemic infection and acute local infection, arguably related to continued i.v. drug abuse, local instability and immunocompromise from chronic hepatitis and malnutrition-induced osteoporosis, as well as inadequate fixation owing in part to short segment, stand-alone anterior fusion may have synergistically contributed to the catastrophic outcome described in this report.

## Conclusion

The dismal outcome of this 59-year patient, secondary to revision fixation for a failed anterior fusion for pyogenic thoracic spondylodiscitis, implies that an inadequate initial treatment modality may lead to a delayed cascade of complicating events with the potential for a lethal outcome. We recommend the more aggressive approach – which is supported by the recent peer-reviewed literature – of a radical surgical debridement associated with a 360° antero-posterior instrumented fusion, for multi-morbid patients suffering from pyogenic spondylodiscitis.

## Consent

Written informed consent was obtained from the patient's relatives for publication of this case report.

## Competing interests

PFS has received speaker's honoraria by Synthes (Paoli, PA) and Stryker Spine (Allendale, NJ). The authors declare no other competing interests related to this case report.

## Authors' contributions

KMB, GEB, EEM, and PFS performed the surgical procedures. MAF and PFS wrote the manuscript. All authors contributed to the revisions of the text and approved the final version of this manuscript.
